# Dual-Functional Coatings for RO Membranes: Optimizing Graphene Oxide and Polydopamine for Fouling and Scaling Control

**DOI:** 10.3390/molecules31101702

**Published:** 2026-05-18

**Authors:** Dana A. Da’na, Mohammad Y. Ashfaq, Woei Jye Lau, Mohammad A. Al-Ghouti

**Affiliations:** 1 Environmental Science Program, Department of Biological and Environmental Sciences, College of Arts and Sciences, Qatar University, Doha P.O. Box 2713, Qatar; dana.adel@qu.edu.qa (D.A.D.); ma1203537@student.qu.edu.qa (M.Y.A.); 2 Advanced Membrane Technology Research Centre (AMTEC), Faculty of Chemical and Energy Engineering, Universiti Teknologi Malaysia, Johor Bahru 81310, Johor, Malaysia; lwoeijye@utm.my

**Keywords:** reverse osmosis, graphene oxide, polydopamine, antifouling, antiscaling, UV treatment

## Abstract

This study reports the development of a novel thin-film nanocomposite (TFN) reverse osmosis (RO) membrane with a surface functionalized using graphene oxide (GO) and polydopamine (PDA). GO was synthesized using a modified Hummers’ method and integrated into a PDA-coated commercial RO membrane. The membranes were treated with UV light for varying durations to enable crosslinking of GO nanoparticles to the membranes. The modified membranes showed improved pure water permeability (PWP) and salt rejection compared to the pristine membrane. The resulting RO membrane, which was exposed to 60 min of UV and contained 0.02 g of GO, achieved the best performance, with a PWP of 23.8 L m^−2^ h^−1^ bar^−1^ and a salt rejection of 96%. Antiscaling and antifouling properties were notably enhanced, as indicated by stable flux under silica scaling and decreased bacterial growth. These results suggest that PDA-GO functionalization is a promising approach for improving membrane durability and efficiency in desalination processes.

## 1. Introduction

Water scarcity remains a critical global issue. According to recent estimates, approximately 2 billion people still lack access to safe drinking water. This problem is particularly severe in arid and semi-arid regions, such as the Middle East and North Africa, where limited renewable freshwater resources exacerbate the crisis [1]. In response, reverse osmosis (RO) desalination has become an increasingly vital technology, now accounting for nearly 70% of the global desalination capacity [2]. 

Thin-film composite (TFC) RO membranes are the benchmark technology in modern desalination systems due to their high selectivity and permeability, as well as their widespread industrial applicability. At the core of RO processes, the TFC membrane offers stable operation and high separation performance and is known as the gold standard for water treatment applications. This can be attributed to their scalability and cost efficiency [3,4]. However, membrane fouling remains a critical limitation to their potential performance. The accumulation of organic matter, inorganic species, and microorganisms on the membrane surface and within its pores increases hydraulic resistance and limits mass transfer, thereby reducing permeate flux under constant pressure. As a result, this leads to higher energy demand, higher maintenance, and shortened membrane lifespan [5].

Fouling can occur in various forms, including organic fouling from natural organic matter, biofouling resulting from microbial growth, and inorganic scaling caused by the precipitation of salts. Hence, these problems can reduce the membrane’s performance while significantly increasing energy demand. Along with the technical consequences, economic issues such as expensive water production, frequent chemical cleaning, and shorter membrane lifespan make the RO technology less competitive [6,7]. Conventional mitigation strategies, such as pretreatment (e.g., coagulation) and chemical antiscalants, are widely employed but often come with limitations, including secondary fouling risks and increased operational complexity [8,9]. 

Recently, membrane surface modifications have emerged as a more sustainable approach to directly enhance the fouling resistance and selectivity of RO membranes, offering the potential to mitigate these issues at their origin. A successful modification, however, must first impart the desired functional properties to the surface while ensuring that it is durably integrated with the underlying membrane to withstand the operational conditions. This study, therefore, was founded on a dual-component strategy intended to create a synergistic composite where the functionality of one material is enabled by the structural role of the other. This has led to the selection of the materials investigated in this study, leading the researchers to explore alternative solutions, with nanotechnology showing considerable promise. Hence, graphene oxide (GO) was chosen as a nanomaterial for membrane surface modification. This is because GO is shown to have exceptional hydrophilicity, high mechanical strength, and rich oxygen-containing functional groups [10,11]. 

As emphasized in the recent literature, integrating GO into membrane surfaces improves surface wettability, enhances water molecule affinity, and reduces fouling propensity by forming a strong hydration layer that acts as a barrier against organic and inorganic foulants. GO also contributes to electrostatic repulsion, aiding in the prevention of charged foulant adhesion. Moreover, the antimicrobial properties of GO further offer protection against biofouling by disrupting bacterial membranes, providing an additional defensive mechanism beyond conventional chemical cleaning [12]. Our previous research has also shown the capability of GO to simultaneously inhibit both biofouling and scaling in RO desalination systems [13].

However, direct incorporation of GO presents challenges, including poor dispersion stability and potential nanoparticle leaching under operational stress. Therefore, polydopamine (PDA) was employed in this research as a versatile intermediate layer that facilitates the uniform dispersion of nanoparticles through ion immobilization and enhances their attachment to the membrane, thereby improving stability [14]. Moreover, the literature strongly supports the role of PDA as a coupling agent that not only secures GO onto the membrane surfaces but also improves membrane hydrophilicity and mechanical robustness [15,16,17]. Because of their hydrophilic catechol groups, strong adhesion to a wide range of surfaces, and amine functionalities, PDA coatings have been extensively investigated as an efficient method to improve the fouling resistance of RO membranes. PDA reduces flux decline during filtration by creating a protective coating on the membrane surface, preventing the attachment of organic foulants. These advancements demonstrate PDA’s promise as an efficient polymer for enhancing the robustness and filtration performance of RO membranes [18,19,20]. 

Moreover, PDA serves as a reactive platform for further chemical modifications, allowing the tailoring of membrane surface properties to target specific fouling mechanisms [21]. When used together, GO and PDA create composite coatings that significantly enhance membrane performance. A study confired that GO-PDA-modified membranes exhibit improved water permeability due to reduced hydraulic resistance and more efficient water molecule transport pathways [15]. Their surface properties, such as hydrophilicity, roughness, and charge, can be tuned by adjusting GO concentrations and UV exposure durations allowing for optimization according to specific operational needs. The hierarchical structures formed by the GO-PDA layer enhance not only permeability but also fouling resistance, owing to the formation of a hydrated and electrostatically repelling surface [22]. Emerging applications, like Janus membranes with asymmetric surface properties, are also benefiting from GO-PDA integration [23,24]. These membranes are being tested for complex separation processes such as oil–water separation and low-pressure desalination, where enhanced selectivity and surface resilience are essential [25]. The combination of GO’s high aspect ratio structure and PDA’s chemical versatility creates a surface that is both robust and functionally dynamic, offering advantages over traditional membrane modification techniques. Most studies still operate at bench-scale, and issues such as cost of materials, scalability of coating methods, and long-term operational stability must be addressed [26].

The above literature briefly explains the importance of GO and PDA in RO membrane fouling research. However, there are no studies that have extensively studied the effect of GO and PDA functionalization to simultaneously control multiple types of biofoulings and scaling. As feedwater contains multiple types of foulants, the performance of RO can be affected by various types of foulants at the same time, and membrane fouling research should target developing RO membranes capable of resisting multiple types of fouling [13]. Moreover, GO and PDA were selected for membrane modification due to their complementary functionalities. GO enhances surface hydrophilicity and contributes to antifouling behavior through its oxygen-containing functional groups, while PDA serves as a robust adhesive layer that improves coating stability and enables uniform deposition of GO. This combination allows the formation of a stable and functional surface with enhanced resistance to fouling [27,28]. 

In this research, we aim to investigate the effect of GO concentrations and UV radiation times on the properties and desalination performance of PDA-coated RO membranes. Furthermore, the GO-PDA-modified RO membrane with optimum desalination performance was also investigated against multiple types of fouling, i.e., silica scaling and biofouling. By establishing the relationship between nanocomposites integration and functional performance, this work provides insight into the underlying mechanisms of nanomaterial-based membrane enhancements and assesses the potential of such strategies for broader application in desalination processes. Hence, this study focuses on surface modification of the commercial RO membrane to provide a practical demonstration of the modification’s potential for large-scale desalination applications.

## 2. Results and Discussion

### 2.1. Effect of Coating on Membrane Desalination Performance

Different GO loadings and UV exposure periods were investigated to determine the optimum GO mass and UV exposure time for the modification of the RO membrane in terms of PWP and solute rejection, as shown in Table 1. The GO masses were varied as 0, 0.005, 0.01, and 0.02 g, while UV exposure duration was 0, 30, and 60 min. For PWP, the experiments were performed with DW, while salt rejection was measured for a 2000 mg/L NaCl solution.

**Figure 1:**
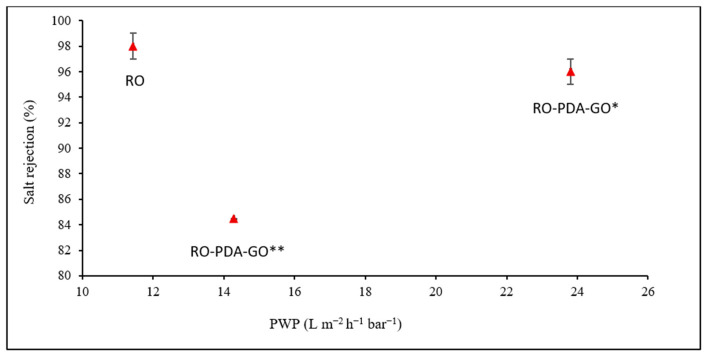
Pure water permeability (L m^−2^ h^−1^ bar^−1^) and salt rejection performance of RO-PDA-GO* and RO-PDA-GO** membranes compared to the pristine RO membrane, highlighting the impact of GO and UV on membrane efficiency. (* 0.02 g of GO, 60 min UV radiation, ** 0.02 g of GO, no UV radiation).

The experimental results demonstrate that surface modification with a GO-PDA nanocomposite significantly enhanced PWP compared to the pristine RO membrane, as shown in Table 1 and Figure 1, providing a direct comparison with a commercial benchmark. The RO-PDA-GO membrane (0.02 g GO and 60 min of UV exposure) showed a marked increase in PWP (23.8 L m^−2^ h^−1^ bar^−1^) compared to the pristine RO membrane. The pristine RO membrane exhibited a lower PWP (11.4 L m^−2^ h^−1^ bar^−1^) and rejection percentage of 98%. Whereas, at GO loading of 0.02 g with 60 min of UV exposure, the PWP increased to 23.8 L m^−2^ h^−1^ bar^−1^, and salt rejection decreased to 96%. This critical observation of a slight decrease in salt rejection is accompanied by an enhancement in water permeability, which may be due to physicochemical changes induced by the surface modification.

The introduction of the GO-PDA layer may swell the polyamide active layer, increasing interstitial voids within the polymer matrix. This can facilitate higher water flux by creating pathways that are large enough to permit small salt ions to pass, thus decreasing overall rejection rates. Furthermore, the deposition process may introduce nanoscale disruptions to the pristine polyamide layer, which could negatively affect its selectivity. Moreover, from an industrial perspective, this reduction presents significant potential for different applications, such as brackish water desalination or the treatment of low-salinity industrial wastewater. The significant improvement in water flux and the reduced need for chemical cleaning due to the enhanced antifouling properties might lead to energy saving and longer membrane lifespan.

As GO loading increases, more carboxyl and hydroxyl groups add to the negative surface charge, boosting the Donnan potential across the active layer, thus restricting co-ion passage (Cl^−^) while allowing counter-ions (Na^+^) through to maintain charge neutrality. When GO nanosheets are well dispersed and crosslinked with PDA under UV light, the surface charge becomes more uniform, reducing defects and leakage zones that could impair rejection [29]. GO also enhances water permeability via increased hydration and electrostatic interactions, while blocking ions, leading to better permeability and salt rejection. UV crosslinking of PDA strengthens the GO nanocomposite by covalently bonding GO to PDA, creating a stable, defect-minimized matrix with improved mechanical stability [30]. Conversely, at the lowest GO loading (0.005 g) without UV treatment, salt rejection drops to 84.5%, worse than the pristine membrane. This indicates that insufficient GO causes uneven surface modification and nanogaps, harming the membrane’s selectivity. The weak surface charge fails to produce strong electrostatic repulsion, lowering rejection. 

AFM results reveal a low roughness of 46.09 nm, suggesting sparse GO deposition, which may only partially fill surface valleys and generate patchy coverage and microdefects that allow salt passage. Similar patterns are seen in other GO membrane studies, where inadequate dopamine leads to defective polyamide layers and lowered rejection [25]. A review on rGO-incorporated membranes reports that overloading (>0.02 wt%) can create defects, reducing salt rejection [31]. Therefore, our AFM and rejection data indicate that minimal GO loading causes patchy coverage, which reduces roughness but does not fully seal the membrane. This creates microdefects that decrease salt rejection.

In contrast, at 0.02 g GO, the increased charge density improves Donnan exclusion, resulting in over 90% salt rejection. Additionally, GO’s hydrophilic nature helps create a stable hydration layer on the membrane surface, which encourages rapid water transport by reducing interfacial resistance and preventing foulant adsorption. With increased GO coverage and effective PDA crosslinking, this hydration layer becomes more continuous and uniform, increasing PWP while maintaining selectivity. The combined effect of electrostatic exclusion and hydration-driven transport accounts for the simultaneous improvements in both salt rejection and water permeability under optimal GO-PDA UV conditions. This underscores the importance of sufficient GO to fully enhance and functionalize the membrane’s selective layer [32]. 

Further increases in GO loading and UV exposure improved desalination performance; for example, the 0.01 g GO samples with 30 min of UV exposure showed a salt rejection rate of 91.1%. This indicates that even modest UV exposure can significantly boost the membrane’s salt rejection capability. The best performance was achieved with 0.02 g of GO and 60 min of UV exposure, confirming that complete surface coverage by GO and thorough crosslinking through UV treatment are crucial for optimal results. Compared to the PDNP-TFN membrane reported by [15], which achieved slightly higher salt rejection (98.4%) but much lower water permeability (4.33 L m^−2^ h^−1^ bar^−1^), the GO-PDA membrane introduced in this work shows a significant permeability improvement (23.8 L m^−2^ h^−1^ bar^−1^).

Khanzada et al. [33] demonstrated that the pristine RO membrane achieved a salt rejection rate of 93–94% when tested with a 2000 ppm NaCl feed solution under an applied pressure of 25 bar. After surface modification with PDA-GO, the membrane exhibited an improved rejection of over 97% under the same operating conditions. However, this increase in rejection was accompanied by a slight decrease in water permeability; the pristine membrane had a flux of approximately 5 L m^−2^ h^−1^ bar^−1^, while the PDA-GO-coated membrane had a flux of about 4.5 L m^−2^ h^−1^ bar^−1^, representing a modest decline of about 10%. This highlights how the surface-localized GO-PDA modification effectively improves water transport while preserving strong salt rejection. It confirms that the synergy between GO’s oxygen-rich nanosheets and polydopamine crosslinking enhances surface hydrophilicity and stabilizes the selective layer, allowing for efficient water flow with minimal impact on ion rejection.

### 2.2. Scaling Test

**Figure 2:**
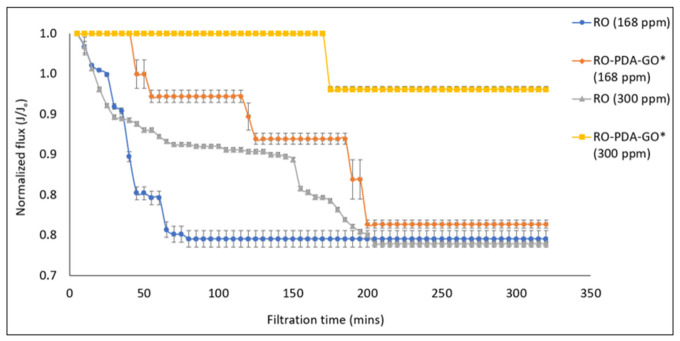
Antiscaling experiments of RO-PDA-GO* compared to the RO membrane under different concentrations of sodium silicate, showing the effect of membrane modification on scaling resistance. Operating conditions: 25 bar, 1 LPM, 2000 ppm NaCl + 168/300 ppm Na_2_SiO_3_, RO-PDA-GO* means RO-PDA modified with 0.02 g GO, and 60 min UV radiation.

Figure 2 shows the normalized flux decline over time for the RO-PDA-GO membrane (0.02 g GO and 60 min of UV exposure) and the pristine RO membrane at varying silica concentrations. The data show the flux behavior of the pristine RO and RO-PDA-GO membrane at silica concentrations of 168 and 300 ppm. The RO-PDA-GO membrane exhibits superior resistance to flux decline, particularly at higher silica concentrations (300 ppm), indicating enhanced antiscaling performance. All membranes showed a flux decline followed by stabilization, suggesting a steady-state condition after the silica deposition. This stabilization indicates the formation of a relatively stable scaling layer, where further deposition is balanced by hydrodynamic shear forces and solubility limitations [34].

At a lower silica concentration of 168 ppm, the RO-PDA-GO membrane maintained an average flux of 23.4 L m^−2^ h^−1^, while at a higher concentration of 300 ppm, it decreased to 19.9 L m^−2^ h^−1^. The higher concentration caused faster saturation and precipitation, leading to increased accumulation of silica on the membrane surface and the formation of a denser scaling layer. Silica differs from conventional scalants such as calcium carbonate or sulfate salts in that it typically forms amorphous, gel-like layers rather than crystalline deposits [35]. The observed decrease in flux at higher concentrations is most likely due to the partial blockage of surface pores and the masking of hydrophilic groups within the PDA-GO coating. As silica deposits grow, they reduce the effective membrane surface area available for water transport and interfere with the hydration layer that normally facilitates rapid diffusion. Additionally, the presence of the scale layer introduces a secondary resistance to mass transfer, which causes further flux reduction.

However, salt rejection performance of the membrane remained consistently high, decreasing only slightly from 98.85% to 98.18% as silica concentration increased. This indicates that the underlying membrane structure and the PDA-GO layer remain functionally intact even when partially covered by scaling. Moreover, the amorphous nature of silica allows permeate flow through microgaps in the scaling layer, which may contribute to sustained rejection despite the reduced flux [36]. The findings from these antiscaling tests highlight a critical advantage of the modified membrane as it exhibits strong resistance to performance degradation under challenging conditions. While a decline in permeability is unavoidable due to physical obstruction, the ability to maintain high salt rejection proves the coating’s durability and functional robustness. This resilience is essential for real-world desalination operations where feedwater compositions fluctuate and often contain high concentrations of species with low solubility, like silica. The PDA-GO modification, by creating a surface that is not only hydrophilic but also chemically stable and electrostatically active, offers a protective mechanism that preserves membrane selectivity under stress, setting the groundwork for longer membrane life and more reliable operation [37].

### 2.3. Biofouling Test

Antibiofouling experiments were conducted to compare the bacterial resistance of the modified RO-PDA-GO membrane (containing 0.02 g of GO and exposed to UV for 60 min) with that of the pristine RO membrane. The OD_600_ value for the filtrate obtained using the RO-PDA-GO membrane, which is 0.912, was slightly higher than that of the pristine membrane (0.879), suggesting an initially lesser antibacterial effect. Consequently, CFU analysis was used to gain a clearer understanding of bacterial attachment, as the RO-PDA-GO membrane showed fewer viable cells, i.e., 1.14 × 10^−4^ cells/mL, compared to 1.2 × 10^−1^ cells/mL for the pristine RO membrane. The difference between the OD_600_ values (0.912 vs. 0.879) and the CFU counts occurs because the OD_600_ measures total turbidity, including both live and dead cells, whereas CFU counts only viable bacteria. The slightly higher OD_600_ for the GO-PDA membrane likely reflects the presence of dead cells or debris released due to GO’s bactericidal action. Despite similar turbidity, the 3-log reduction in CFU confirms a significant decrease in viable bacteria. This reduction in bacterial growth demonstrates the antibacterial activity of the GO-modified surface.

This finding aligns with the results of Khanzada et al. [38], who demonstrated that the PDA-GO-modified membrane significantly decreased bacterial viability compared to the pristine RO membrane. Additionally, CFU analysis showed a drastic reduction in viable *Bacillus cereus* on the modified surface (12% viability vs. 99.9% on the pristine membrane), confirming its strong bactericidal effect. Although surface hydrophilicity was not directly measured, FTIR results confirmed the presence of oxygen-containing functional groups, which are known to enhance membrane hydrophilicity. The performance is likely associated with the negatively charged surface and the presence of oxygen-containing functional groups introduced by the GO nanosheets, as supported by FTIR analysis, which creates an electrostatic barrier that limits bacterial adhesion and biofilm formation [39]. The oxygen-containing functional groups are known to promote hydration layer formation, physically preventing microbial attachment [6]. 

The UV crosslinking also enhances the stability of the PDA-GO layer, maintaining surface properties and functionality under operational conditions. Similarly, Ref. [40] also reported that GO has inherent antimicrobial properties that effectively reduce microbial growth in polymeric membranes. This ability is due to GO’s capacity to disrupt bacterial cell membranes and inhibit biofilm formation, which corresponds with our CFU results showing reduced bacterial viability. Collectively, these effects explain the significant reduction in CFU and demonstrate how the modified membrane resists bacterial colonization through both physical disruption and electrostatic repulsion.

Prolonged filtration tests are vital for evaluating long-term stability and fouling resistance of RO membranes under realistic operating conditions. By regularly exposing membranes to feed waters containing common foulants and scaling agents, these tests provide insights into the durability of surface modifications. They simulate real-world use scenarios, helping researchers understand membrane performance over time. Continuous testing under typical industrial or municipal water treatment conditions assesses both initial effectiveness and durability against fouling and scaling. These tests also evaluate the performance of surface modifications in enhancing fouling resistance, aiding the development of more reliable membrane technology. This understanding is crucial for optimizing performance, minimizing maintenance costs, and enhancing RO efficiency.

The existing literature shows that GO, PDA, and combined GO-PDA surface modifications can greatly improve the long-term stability of RO membranes when facing fouling and scaling challenges. The antifouling properties of polyamide RO membranes (Toray UTC73HA, Tokyo, Japan) were enhanced by surface modification with GO using the Langmuir–Blodgett (LB) method [41]. GO nanoflakes were aminated with ethylenediamine for covalent bonding, and the membrane’s carboxyl groups were pre-activated with EDC/NHS for durable adhesion. GO was assembled at the air–liquid interface under controlled pressures (10–40 mN/m) and then transferred onto the membrane. Filtration tests with brackish water (2000 mg/L NaCl, 200 mg/L bovine serum albumin, BSA) operated at 12 bars with periodic compaction at 18 bars. The water permeance of the modified membrane slightly decreased from 2.70 to 2.28 L m^−2^ bar^−1^ h^−1^, but NaCl rejection improved from 95.2% to 98.1% due to effective defect sealing. It also showed a 52% lower flux decline ratio (FDR) during BSA fouling and an increased flux recovery ratio (FRR) of 87%, compared to 65%, indicating better fouling reversibility. Characterization confirmed a smoother, more hydrophilic surface (contact angle decreased from 67° to 55°), which remained stable after three fouling–cleaning cycles, every 240 min. Additionally, GO-coated membranes maintained stable performance under long-term cross-filtration for more than 30 h, as reported by Zhong et al. [42], achieving over 98% NaCl rejection with high flux recovery rates (>90%) after BSA fouling, supported by a PDA interlayer that prevents GO re-dispersion. Overall, these findings demonstrate that with proper stabilization via crosslinking, protective PDA layers, or layered architectures, GO and PDA modifications can sustain high salt rejection, flux stability, and fouling resistance during extended or repeated use, which is essential for practical long-term desalination applications.

### 2.4. Characterization of the Prepared Membranes

**Figure 3:**
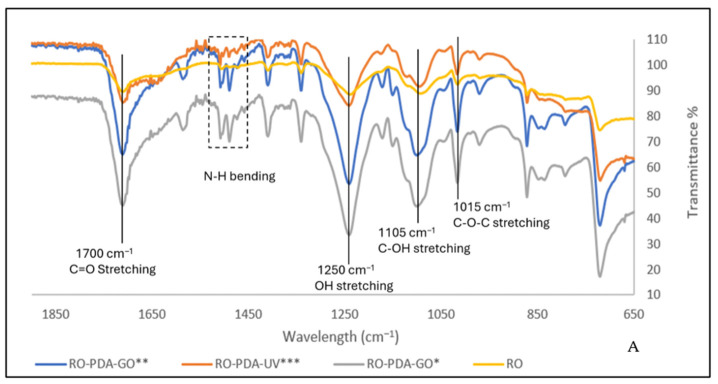

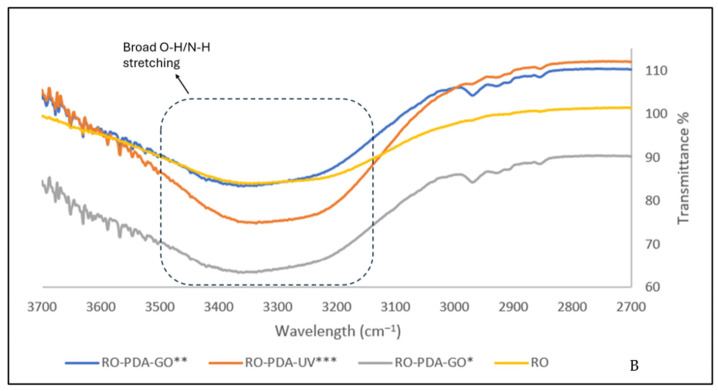
FTIR spectra of pristine RO, RO-PDA-GO*, RO-PDA-GO**, RO-PDA-UV*** membranes before antiscaling test illustrating the changes in chemical structure due to different GO loading and UV exposure. (**A**) spectra range from 600 to 2000 cm^−1^, (**B**) spectra range from 2700 to 3700 cm^−1^. (* 0.02 g GO, UV 60 min, ** 0.02 g GO, no UV, *** no GO, UV 60 min).

The FTIR findings support the observed enhancements in the RO-PDA-GO membrane (0.02 g GO and 60 min of UV exposure) performance, as shown in Figure 3. The chemical integration of PDA and GO, supported by UV, has resulted in a chemically stable, hydrophilic, and functionally enhanced membrane surface. This directly correlates with enhanced water flux, high salt rejection, and better biofouling and inorganic scaling resistance. The pristine RO membrane showed peaks at around 1650 cm^−1^ and 1540 cm^−1^, corresponding to C=O stretching and N–H bending, respectively. After PDA-GO modification, broadened bands in the 3200–3400 cm^−1^ range, attributed to O–H and N–H stretching vibrations, were observed, reflecting increased surface hydrophilicity and the presence of hydrogen-bonding sites. These peaks are attributed to the combined presence of hydroxyl groups from GO and the amine and catechol functionalities of PDA. In addition, a distinct peak observed around 1450–1540 cm^−1^ aligns with aromatic ring vibrations of PDA’s catechol groups, confirming its successful integration. Together, these bands reflect the enhanced hydrophilicity and chemical complexity introduced by the GO-PDA coating.

These results are aligned with Iqbal et al. [43] who suggested that the excessive existence of hydroxyl groups in the 3200–3400 cm^−1^ range caused more broadening and intensity of the peaks. The RO-PDA-GO membrane showed broader absorption in this region compared to the pristine RO membrane and RO-PDA-GO without UV and RO-PDA under 60 min UV, indicating a denser network of hydrophilic groups, specifically, which caused reinforced membrane stability through π-π interactions and potential covalent bonding with PDA.

**Figure 4:**
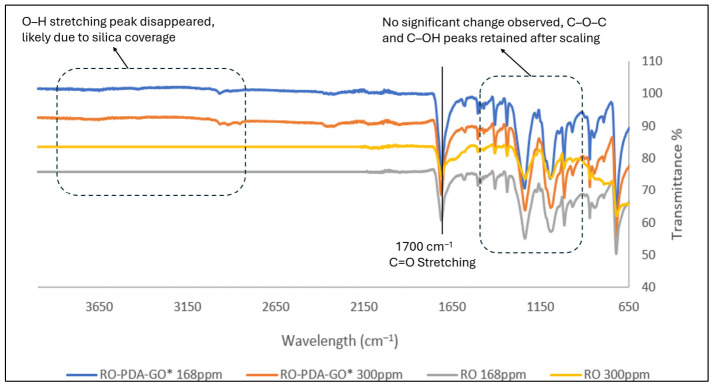
FTIR spectra of pristine RO membrane and RO-PDA-GO* membrane after antiscaling test with 168 ppm and 300 ppm. (* 0.02 g, UV 60 min).

Incorporating GO into the RO-PDA membrane is strongly supported by distinct peaks observed in the FTIR spectra around 1000–1100 cm^−1^, which were not present on the RO surface as shown in Figure 4. These peaks represent the stretching vibrations of C–O–C (ether) and C–OH (hydroxyl) groups, characteristic of oxygen-rich functional groups in GO. These bands were completely absent in the pristine RO membrane, indicating it lacks such oxygen-functionalized components. The presence of these functional groups in the RO-PDA-GO membrane confirms the successful chemical integration of GO nanosheets into the polyamide matrix during modification. These functional groups significantly enhance the membrane’s hydrophilicity, thereby increasing water permeability and reducing the adhesion of hydrophobic foulants. They also create polar sites that facilitate strong interactions with water molecules through hydrogen bonding, optimizing water transport. Additionally, they serve as reactive anchors for PDA crosslinking, especially under UV exposure, promoting interfacial polymerization and enhancing structural stability. Therefore, these peaks not only confirm the successful incorporation of GO but also explain the improved long-term antiscaling and antifouling performance of the RO-PDA-GO membrane compared to the pristine membrane. Similarly, it has been found that these bands are associated with enhanced surface reactivity and potential sites for crosslinking and resistance to fouling [43,44].

The spectral shifts and intensity variations across the 900–1600 cm^−1^ region further suggest strong intermolecular interactions that help reduce structural defects and support membrane performance during desalination and fouling scenarios due to the enhanced mechanical integrity, compactness, and cohesiveness of its surface layer. Deeper insights into the structural modification and the chemical interactions in the RO-PDA-GO membrane could be discussed in terms of the spectral shifts and intensity variations observed across specific peaks within the 900–1600 cm^−1^ region. The presence of a peak near 1030 cm^−1^ corresponds to C–O–C stretching vibrations, confirming the existence of ether groups, while the presence of hydroxyl functional groups is shown on the peak around 1105 cm^−1^ assigned to C–OH stretching. Both peaks confirm that they were introduced by GO. The presence of PDA resulted in the presence of aromatic ring vibration, as confirmed by the peak at 1450 cm^−1^. Furthermore, in the RO-PDA-GO membrane, a slightly increased intensity near 1540 cm^−1^ corresponding to N–H bending and C–N stretching was observed, which indicates the improved hydrogen bonding as well as the crosslinking between the polyamide matrix, PDA, and GO. Moreover, it has been confirmed that the dense surface integration of the GO-PDA membrane due to the presence of such bands in this region [45]. The molecular interactions between GO, PDA, and polymer matrix facilitated better stability in terms of operation under fouling conditions as well as improved mechanical robustness.

FTIR analysis also showed chemical changes in the morphology of the membrane after exposure to silica scaling (Figure 4). A key observation was the disappearance of a broad absorption peak above 3000 cm^−1^, which could be due to the hydroxyl groups present on the surface, suggesting interaction or coverage by deposited silica. This disappearance of the broad O–H stretching band after exposure to silica-rich feedwater (Figure 4) indicates that the surface hydroxyl groups were either physically masked or involved in interactions with silica deposits. Specifically, these interactions likely involve hydrogen bonding between the hydroxyl groups of GO and the silanol (Si–OH) groups of amorphous silica. These results are consistent with those of [46] as their study also shows characteristic peaks near 3400 cm^−1^ and 1740 cm^−1^, corresponding to O–H and C=O stretching vibrations from carboxylic acid groups. However, when GO is incorporated into a silica matrix, these oxygen-containing functional group peaks largely disappear, suggesting chemical interactions between GO and the silica network, likely involving bonding between GO functional groups and silanol (Si–OH) groups. Additionally, the presence of GO alters the intensity of Si–O–Si and Si–OH bands in the FTIR spectrum, indicating that GO–silica interactions occur during the formation or deposition process. These changes directly support the observed improvements in hydrophilicity, antifouling, and antiscaling properties, offering a molecular-level explanation for the enhanced performance seen in permeability, salt rejection, and resistance to biofouling and inorganic fouling.

**Figure 5:**
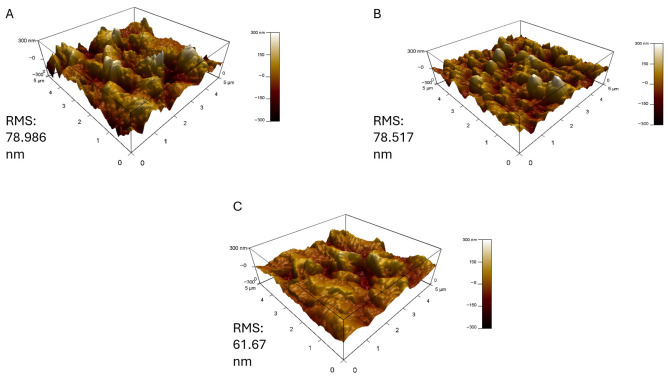
Microscopic characterization of RO-PDA-GO* membrane with AFM (**A**) before antiscaling test, (**B**) after antiscaling with 168 ppm silica salt, (**C**) after antiscaling with 300 ppm silica salt. (* 0.02 g, UV 60 min).

**Figure 6:**
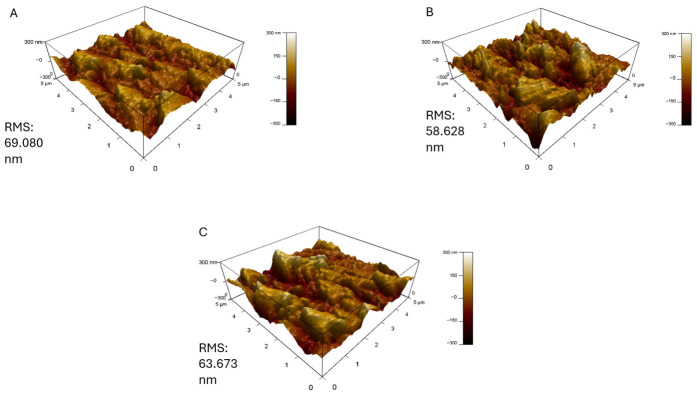
AFM characterization of pristine RO membrane for comparison with the surface changes in the modified membrane, (**A**) before antiscaling test, (**B**) after antiscaling with 168 ppm silica salt, (**C**) after antiscaling with 300 ppm silica salt.

The AFM analysis of the RO-PDA-GO membranes provides critical insights into the morphological changes induced by the GO coating and its interaction with silica scaling, as shown in Figure 5. The pristine RO membrane exhibited a relatively smooth surface with a root mean square (RMS) roughness of 69.08 nm (Figure 6). However, after modification, the RO-PDA-GO membrane displayed a higher RMS roughness of 78.99 nm, as a result of GO introduction as well as the formation of a crosslinked PDA network. According to Samadi et al. [47], though increased surface roughness is often associated with higher levels of fouling, in this case, it reflects the well-organized hierarchical nanostructure formed by the PDA-GO composite, which enhances both functionality and resistance to fouling. These results are correlated with the results obtained by [48] where the decrease or increase in surface roughness with the addition of GO-based nanocomposite depends on the concentration of the nanofiller and the compatibility of the GO polymer. As a result of the better dispersion and compatibility, moderate loading (e.g., 0.085 wt%) of graphene oxide–chitosan nanocomposite (GO-CH) produced smoother membrane surfaces in their investigation, while excessive filler content (e.g., 0.136 wt%) resulted in nanoparticle aggregation and noticeably increased roughness. The introduction of GO and PDA contributed to a more textured membrane surface, effectively increasing the surface area and enhancing hydrophilicity.

Upon exposure to silica-rich feedwater, the AFM images revealed distinct scaling patterns dependent on silica concentration. At 300 ppm, the membrane surface developed dense, irregular deposits that correlated with the significant flux decline to 20.72 L m^−2^ h^−1^. The three-dimensional AFM topography showed localized clusters of silica aggregates, suggesting heterogeneous nucleation and growth on the nanocomposite surface. The increased surface roughness, observed both before and after silica exposure, aligns with the roughness-driven structural alterations reviewed by Samadi et al. [47] where surface-modified membranes exhibited increased topographic variation that contributed to overall stability under environmental and chemical stress. This observation aligns with the FTIR data, where the disappearance of surface hydroxyl peaks indicated physical coverage by silica rather than chemical bonding. At the lower silica concentration (168 ppm), the AFM images displayed more uniform and sparse scaling, consistent with the membrane’s ability to maintain stable flux (18.57 L m^−2^ h^−1^). The difference in scaling morphology between the two concentrations shows the protective role of the PDA-GO layer during silica deposition.

The relationship between surface morphology and antibiofouling performance can be inferred from the AFM results. The increased roughness of the modified membrane (RMS = 78.99 nm) compared to the pristine RO limits the bacterial adhesion, leading to decreased biofouling, which creates a surface topography that is too irregular for bacterial cells to establish strong contact points. The decrease in roughness with increasing silica concentration supports the idea that the scaling layer forms a physical barrier that masks the underlying nanostructure of the PDA-GO coating. While the surface becomes smoother, the GO-PDA layer still contributes to maintaining membrane performance by providing hydrophilicity and electrostatic repulsion. These results align with Zhao et al. [49] who demonstrated that for hydrophilic membranes, increasing surface roughness enhances the interfacial hydration repulsion barrier, reducing fouling by reducing the adhesion of foulants. Moreover, these results are supported by the FTIR results, where the oxygenated functional groups present on the GO surface enhance hydrophilicity and introduce a negative surface charge, explaining the reduced bacterial adhesion.

**Figure 7:**
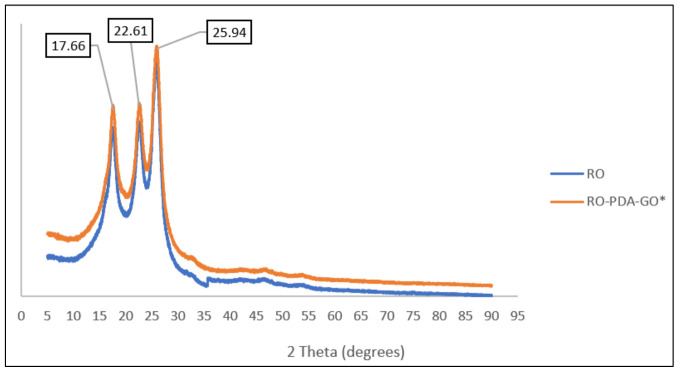
XRD spectra of the precipitate confirms the formation of silica crystals (168 ppm) on RO-PDA-GO* and pristine RO membrane. (* 0.02 g, UV 60 min).

The XRD analysis revealed distinct crystallographic features that support the structural findings from FTIR and AFM. The pristine RO membrane displayed a broad amorphous peak around 2θ ≈ 20–25°, as shown in Figure 7. In contrast, the RO-PDA-GO membrane exhibited sharp peaks approximately at 2θ ≈ 10–12° and 26–28°, which correspond to the (001) and (002) planes of GO, confirming successful integration of GO nanosheets into the membrane matrix. These results align with Liu et al. [50] who reported an XRD diffraction peak of GO-PDA around 12.0° corresponding to the (001) crystal face of the crystalline–hexagonal GO structure, indicating successful functionalization of GO with PDA. These crystalline features coexist with the amorphous polymer network, forming a dual-phase nanocomposite structure. It has been reported that a lower angle shift of the diffraction peak indicated the successful oxidation of GO due to the introduction and intercalation of carboxyl and hydroxyl oxygen groups [51].

This crystalline–amorphous interaction aligns well with FTIR results, where O–H and N–H stretching bands (3200–3400 cm^−1^) indicate the presence of oxygenated functional groups introduced by GO. Additionally, the nanoscale roughness observed in AFM scans (RMS ≈ 78.99 nm) likely results from GO crystallites spreading across the membrane surface, enhancing the surface area. The peaks at 17.9°, 23°, and 26°, linked to the (010), (−110), and (100) planes of silica, show the formation of crystalline SiO_2_ during scaling tests [52]. These results are consistent with Li et al. [53] who reported that adding crystalline nanoparticles to the polymer membrane caused peaks to appear over the amorphous halo of the polymer base, leading to increased mechanical strength, porosity, and surface roughness.

The improved performance of the RO-PDA-GO membrane can be attributed to the synergistic interaction between GO and PDA, stabilized through UV-assisted crosslinking. While both GO and PDA are hydrophilic, their contributions to the antibiofouling mechanism can be distinguished. PDA’s primary role is to act as a robust molecular adhesive, creating a foundational layer that firmly anchors the subsequent GO nanosheets to the polyamide surface via π-π interactions and hydrogen bonding. Once anchored, the GO nanosheets form the dominant antifouling barrier at the membrane–water interface. Their exceptional hydrophilicity and negative surface charge, derived from abundant oxygen-containing functional groups (confirmed by FTIR in Figure 3 and Figure 4), create a strong hydration layer and electrostatic repulsion that minimizes foulant adhesion.

Therefore, the synergy arises from PDA acting as the critical binder that enables the stable and effective presentation of the antifouling GO nanosheets. UV irradiation enhances this stability by promoting covalent bonding between PDA’s catechol/amino groups and GO’s epoxy/hydroxyl sites, forming a defect-minimized network that resists nanoparticle leaching under crossflow conditions [53]. Furthermore, XRD analysis as shown in Figure 7, supports the successful incorporation and structural alignment of GO within the membrane matrix. This combined mechanism results in a nanocomposite coating that enhances water flux and improves durability during extended filtration tests.

Although the GO-PDA surface modification process demonstrated promising improvements at the laboratory scale, further attention is required for its scalability and practical integration into industrial membrane production lines. It has been mentioned that the main advantage of GO modification is its compatibility with conventional coating methods, such as dip-coating, vacuum filtration, and layer-by-layer assembly, which can be incorporated into current TFC membrane fabrication [54]. Further advantages are the ability to perform the GO coating under mild temperatures and ambient conditions. However, large-scale implementation must address practical challenges, including achieving uniform coating coverage across full-size membrane sheets or spiral-wound modules, controlling GO layer thickness, and ensuring stable adhesion under high-pressure crossflow to prevent GO nanoparticle leaching during long-term operation. Another important consideration is the additional UV-C irradiation step used to crosslink the PDA-GO layer, which would need to be integrated into continuous membrane production without significantly increasing energy costs or slowing down manufacturing throughput. Despite these challenges, the mild chemical conditions, low nanofiller loading, and simple coating procedures strongly support the feasibility of extending this modification approach to pilot or industrial scales, provided that future work demonstrates stable coating performance, acceptable fabrication costs, and reliable module integration under real desalination operating conditions [55].

## 3. Methodology

### 3.1. Synthesis of GO

**Figure 8:**
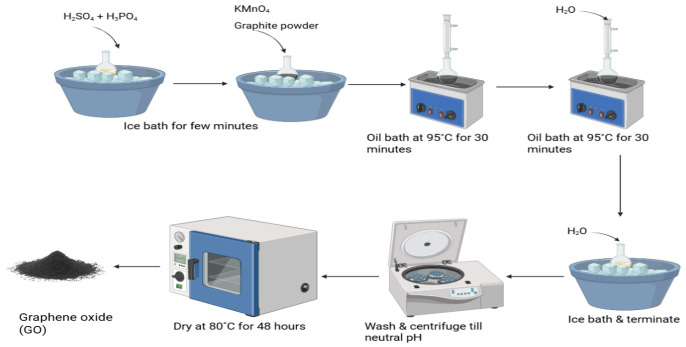
Schematic illustration of Hummers’ method for graphene oxide preparation.

Briefly, GO was prepared based on a modified Hummers’ method as reported in the work of [56]. Figure 8 shows the process flow of GO synthesis. Briefly, GO was prepared by oxidizing graphite using a mixture of sulfuric acid (H_2_SO_4_) and phosphoric acid (H_3_PO_4_), which was cooled in an ice bath for a few minutes. This was followed by the addition of graphite powder and potassium permanganate (KMnO_4_) to initiate the oxidation process. After that, the reaction is heated in an oil bath at 95 °C with gradual additions of water to maintain the oxidation process for 30 min. The mixture was cooled again in an ice bath and diluted further to stop the reaction. The resulting suspension is washed and centrifuged repeatedly until a neutral pH is reached, then dried at 80 °C to obtain the final graphene oxide powder.

### 3.2. Modification of Commercial RO Membrane with GO-PDA

The SWRO membrane (SW30XLE) was selected due to its well-defined polyamide (PA) selective layer and high salt rejection, providing a robust and consistent baseline for evaluating the impact of surface modification. The modification of the commercial RO membrane (energy-saving polyamide (ESPA)) thin-film composite (TFC) RO membranes (SW30XLE, which was obtained from Dow Filmtec/Edina, MN, USA) with graphene oxide–dopamine hydrochloride (GO-PDA) was carried out as follows: The required mass of GO (0, 0.005, 0.01, and 0.02 g) was weighed and added, respectively, to 15 mM of tris buffer. The prepared GO buffer solution was sonicated using an Elmasonic Select 100 ultrasonic bath (37 kHz, 150 W-Elma Schmidbauer GmbH, Singen, Germany) for 60 min until the GO was well dispersed in the buffer solution. The absence of sediment and a stable, dark brown color visually ensured uniformity of dispersion. After that, the GO-buffer solution was centrifuged, and dopamine hydrochloride (2 g/L) was added to the obtained GO suspension. After that, the pH was adjusted to 8.5. The concentration of dopamine hydrochloride (2 g/L) was selected based on the protocol which is widely used in surface modification of RO and ultrafiltration membranes [44,57].

The prepared GO-PDA suspension was exposed to UV (UV-C irradiation, UVG–11 Compact UV lamp from John Morris Scientific Pty Ltd., Chatswood, NSW, Australia, 254 nm wavelength and 4 W, 0.12 A). The sample was irradiated at a distance of 0.83 cm from the lamp for 180 min before being poured into the commercial RO membrane and then exposed again to UV for the required times (0, 30, and 60 min), as longer UV exposure can cause damage to the membrane. The GO-PDA suspension was exposed to UV for 180 min, which was the minimum required time to observe a noticeable change in the suspension color (darkening). This color change indicates progress in the photochemical process, and no further change occurred when time was extended. The UV-assisted crosslinking of the GO-PDA layer was adopted because it enhances coating stability and reduces nanoparticle leaching risks, providing a promising route toward commercial viability. The incorporation of UV treatment not only strengthens adhesion but also improves the chemical resilience of the modified surface under real-world desalination conditions [58,59]. After UV-assisted crosslinking of the GO-PDA layer, the suspension was discarded, and the membrane was placed in an oven at 40 °C for 30 min. Finally, the membrane was washed with distilled water (DW) and stored in DW for further use and characterization.

### 3.3. Measuring Desalination Performance of Pristine RO and GO-PDA-Modified RO Membrane

The experiments were carried out under fixed operating conditions of temperature (25 °C), flow rate (1 L/min), and transmembrane pressure (25 bars) based on previous literature [60]. The experiments were investigated at two different silica concentrations (168 ppm and 300 ppm) in terms of pure water flux and salt rejection (2000 ppm NaCl). All samples were run for 2 h with DW and 6 h with the prepared feed water solution. After that, the RO membrane was cut to the required size, washed with distilled water, and kept in DW for 24 h. Then, 6 L of sodium metasilicate of the required concentration (168/300 ppm) and sodium chloride (2000 ppm) solution was prepared by adjusting the pH to 6.7. The membrane was placed in the cell in the crossflow filtration system, and the tank was filled with the prepared feedwater. The crossflow filtration system was started and adjusted to the required flow rate and pressure.

Equations (1) and (2) were used to measure pure water permeability, PWP (L m^−2^ h^−1^ bar^−1^) and salt rejection, R (%), respectively. PWP was used to evaluate the intrinsic hydraulic performance of the membrane without the effect of osmotic pressure. Whereas desalination performance was evaluated using NaCl rejection under saline conditions.

(1) $$PWP=\frac{Q_{p}}{∆P\times A}$$

(2) $$R=\frac{\left(C_{f}-C_{p}\right)}{C_{f}}\times 100$$

where for Equation (1), Q_p_ is the permeate flow rate (L h^−1^), A is the effective membrane area (m^2^), and ΔP is transmembrane pressure (bar). In Equation (2), C_f_ and C_p_ are the conductivity of feedwater and the conductivity of permeate, respectively. The conductivity was measured using an electrical conductivity meter (HQ440d, multi, HACH, London, UK).

### 3.4. Scaling Experiments

To assess the antiscaling properties of RO-PDA-GO membranes, silica was selected as a model scalant with two concentrations: 168 and 300 mg/L. A sodium metasilicate (Na_2_SiO_3_) solution was prepared and combined with a NaCl solution (2000 mg/L), followed by adjusting the pH to 6.7. The membrane (either pristine RO or RO-PDA-GO) was positioned in the cell of a crossflow filtration system, and the tank was filled with the prepared feedwater. Scaling experiments were conducted at a pressure of 25 bar, a flow rate of 1 LPM, and a temperature of 25 °C. These experiments were carried out continuously for 6 h after an initial 2 h compaction period using DW.

### 3.5. Biofouling Experiments

The biofouling experiments were carried out following the protocol described elsewhere [8]. The RO-PDA-GO (0.02 g GO and 60 min of UV exposure) and pristine RO membrane were disinfected with 70% ethanol for 30 min, followed by washing with autoclaved water to remove any excess ethanol. Bacterial inoculum from *Bacillus cereus* culture was prepared with an initial optical density (OD_600_) of 0.1 and added to a 50 mL centrifuge tube containing sterile LB medium. The membrane was cut into squares of 1 × 1 cm and added to the centrifuge tubes that contained the growth medium. The tubes were incubated in an incubator shaker for 18 h at 30 °C. The number of bacterial cells at *t* = 0 and *t* = 18 h was obtained. The bacteriostasis rate was calculated using Equation (3).

(3) $$Bacteriostasis\;rate=\frac{n_{0}-n_{1}}{n_{0}}\times 100$$

where n_0_ is the number of colonies on the plates treated with the pristine RO membrane, while n_1_ is the number of colonies on the plates treated with the RO-PDA-GO membrane.

Freshly cultivated isolates were initially extracted in LB to create an inoculum. After that, 25 mL of LB medium was placed in centrifuge tubes with the required inoculum volume to achieve an OD_600_ of 0.1. Then, the liquid cultures were incubated in a shaking incubator at 30 °C and 150 rpm. Using a UV/Vis spectrophotometer to measure the optical density (OD_600_) and by spreading serial dilutions on LB solid plates to determine the colony-forming unit (CFU), the growth of the strains was determined. The slope of the exponential phase of the bacterial growth curve was used to determine the specific growth rates. CFU through spreading serial dilutions on LB solid plates was also carried out using the following equation:

(4) CFUmL=Number of colonies×dilution factorVolume of culture plate

## 4. Conclusions

This study demonstrated the surface modification of commercial RO membrane with a graphene oxide–polydopamine (GO-PDA) nanocomposite. The membrane fabricated with 0.02 g of GO and 60 min of UV-C exposure, exhibited remarkable improvements in PWP and salt rejection compared to the pristine membrane, confirming the efficacy of the combined chemical and photochemical treatment. The modified RO-PDA-GO membrane exhibited significant improvements in desalination performance, with PWP of 23.8 L m^−2^ h^−1^ bar^−1^ and salt rejection of 96%, compared to the pristine membrane’s 11.4 L m^−2^ h^−1^ bar^−1^ and 98%, respectively. Under silica-rich feedwater, the RO-PDA-GO (0.02 g GO and 60 min UV) membrane showed superior antiscaling behavior, in which it maintained high performance even at 300 ppm silica, with flux values averaging 20.72 L m^−2^ h^−1^ and salt rejection above 98%. Based on the antibiofouling experiments, where the bacterial viability was reduced by >90% compared to the pristine membrane, confirming the successful GO-PDA coating, which provided a bacterial-resistant surface as well as enhanced hydrophilicity, surface charge effects, and nanoscale topography. Characterization through FTIR, AFM, and XRD confirmed the successful chemical integration and morphological alterations responsible for the improved performance. The findings highlight the potential of GO-PDA functionalization not only to boost membrane operational efficiency but also to extend membrane lifespan under realistic desalination conditions. This approach offers a promising pathway toward the development of next-generation membranes that are simultaneously resistant to both fouling and scaling, addressing critical challenges in sustainable water treatment technologies. Although the GO-PDA functionalization shows significant potential, it is important to point out that these findings were achieved using the commercial Dow Filmtec SW30XLE RO membrane. As a result, the effectiveness and optimal parameters of this modification process could vary for other RO commercial membranes due to the differences in membrane structure, composition, and surface properties.

## Figures and Tables

**Table 1:** Obtained results of PWP and salt rejection (2000 mg/L NaCl) of membranes at 25 bar and 1 LPM after investigating different GO loadings under different UV radiation durations

Exp. #	GO Mass (g)	UV Duration (min)	PWP (L m^−2^ h^−1^ bar^−1^)	Salt Rejection (%)	Flux (L m^−2^ h^−1^)
1	0	30	12.4	84.5	18.57
2	0	60	14.3	84.7	21.4
3	0.005	0	12.4	84.5	18.57
4	0.005	30	13.3	89.3	20
5	0.005	60	13.3	84.6	20
6	0.01	0	13.3	85.0	20
7	0.01	30	16.2	91.1	24.3
8	0.01	60	18.1	92.5	27.1
9	0.02	0	14.3	82.8	21.4
10	0.02	30	16.2	88.4	28.3
11	0.02	60	23.8	96.0	35.7
RO pristine membrane	-	-	11.4	98.0	17.1

## Data Availability

The original contributions presented in this study are included in the article. Further inquiries can be directed to the corresponding authors
